# Rethinking *Mycoplasma hyopneumoniae* adhesion: a glycocalyx-mediated infectious niche model in porcine respiratory infection

**DOI:** 10.1186/s13567-026-01836-5

**Published:** 2026-10-02

**Authors:** Yuan Zhao, Anxiong Huang, Xingchi Kan, Wei Wang, Suhua Guan, Xiaochun Tang, Guoqing Shao

**Affiliations:** Xianghu Laboratory, Hangzhou, 311231 China

**Keywords:** *Mycoplasma hyopneumoniae*, porcine respiratory infection, glycocalyx, glycosaminoglycans, adhesins, plasminogen, infectious niche

## Abstract

*Mycoplasma hyopneumoniae* (Mhp) is the primary etiological agent of porcine enzootic pneumonia and establishes infection through close association with the respiratory ciliated epithelium. Classical models of Mhp colonization have focused mainly on specific interactions between adhesins, particularly P97, and ciliary receptors. Although this framework has provided important insights into ciliary attachment, increasing evidence indicates that Mhp adhesion involves multiple surface proteins and host ligands, including sulfated glycosaminoglycans (GAGs), fibronectin, plasminogen (Plg) and extracellular actin. In this review, we synthesize current evidence and propose a glycocalyx-mediated infectious niche framework for early Mhp colonization. Within this framework, sulfated glycans within the respiratory glycocalyx may contribute to bacterial positioning at the airway interface, while multiligand adhesin interactions may contribute to attachment and Plg-binding surface proteins may facilitate host-factor recruitment. Among these interactions, plasminogen recruitment provides a potential link between adhesion and local interface modification, including possible extracellular matrix remodeling and epithelial injury. Other processes, including potential GAG-mediated local retention, host glycan-dependent variation in attachment and glycocalyx remodeling, remain hypotheses requiring further validation. By distinguishing experimentally supported interactions from mechanistic inferences, this review presents Mhp colonization as a dynamic host-pathogen interface process rather than a simple adhesin-receptor event. This framework provides a basis for future investigation of early colonization mechanisms and anti-adhesion strategies in porcine enzootic pneumonia.

## Introduction

*Mycoplasma hyopneumoniae* (Mhp) is the primary etiological agent of porcine enzootic pneumonia, a chronic respiratory disease that reduces growth performance, increases medication costs and causes substantial economic losses in swine production. Mhp initiates infection through close association with the porcine respiratory ciliated epithelium, and adhesion to the airway surface is widely regarded as a critical early step in colonization and disease development [1, 2]. Early in vivo ultrastructural studies provide important spatial context for this interaction. Electron microscopy of infected porcine respiratory tissues showed that Mhp organisms occupy the ciliary compartment, although their precise localization differed among studies. It was observed organisms located deep among respiratory cilia, separated from the epithelial surface by ruthenium-red-positive capsule-like material and fibrillar structures [3]. Blanchard et al. reported Mhp predominantly associated with remaining cilia tips, with fibrillar structures connecting organisms or linking Mhp to cilia, while direct contact with epithelial plasma membrane or microvilli was not observed [4]. Immunogold electron microscopy further demonstrated that P102-associated material was present on Mhp and swine cilia during experimental infection [5]. Together, these observations indicate that Mhp colonization occurs within a structurally complex ciliary interface and may involve host- and bacterial-derived extracellular components rather than direct interaction with an exposed epithelial membrane receptor alone. However, the molecular identity of these intervening structures remains unresolved.

Early studies placed particular emphasis on interactions between mycoplasmal adhesins, especially P97, and receptors on respiratory cilia. This foundational adhesin-receptor framework has provided important insights into Mhp epithelial tropism and host specificity [6]. However, subsequent studies identified additional Mhp surface proteins and host ligands, indicating that colonization cannot be fully explained by a single adhesin-receptor pair. Variation in adhesin sequences and repeat regions has been documented among Mhp strains [7] and may contribute to strain-dependent differences in host interaction properties. These observations support a broader view of Mhp adhesion as a multicomponent host-pathogen interaction process.

The respiratory glycocalyx represents a potential interface for such interactions. This glycan-rich environment contains mucins, proteoglycans, glycolipids and GAGs that regulate molecular accessibility and ligand presentation at the airway surface [8–12]. For Mhp, several surface adhesins and processed fragments can interact with sulfated GAGs, fibronectin and Plg, suggesting that adhesion may involve coordinated engagement of multiple host-associated ligands within a multicomponent interface [13–24]. Based on these observations, this review proposes a glycocalyx-mediated infectious niche model for early Mhp colonization, in which glycocalyx-associated interactions may contribute to bacterial enrichment, adhesin-mediated stabilization and host-factor recruitment.

Because relatively few studies have directly examined the earliest events of Mhp colonization at the native porcine airway surface, this review adopts an evidence-informed, hypothesis-generating perspective. The proposed framework is intended to organize current evidence and identify unresolved mechanistic connections rather than represent an established in vivo infection pathway. The review focuses on molecular interactions occurring at the ciliated airway interface, including glycocalyx-associated attachment, multivalent adhesin-ligand interactions and host-factor recruitment. Although other airway cell populations and immune responses may influence infection progression, detailed mechanisms involving these components are beyond the scope of this review. Throughout the manuscript, experimentally supported interactions are distinguished from mechanistic inferences and exploratory hypotheses to provide a framework for investigating early Mhp colonization.

## The porcine respiratory glycocalyx as a dynamic initial interface for Mhp

After entering the respiratory tract, Mhp encounters a heterogeneous ciliary interface composed of mucus, the periciliary compartment, membrane-tethered mucins, proteoglycan-associated glycans and glycolipids. These components form an interconnected environment rather than sharply separated layers, and in vivo studies have localized Mhp at different positions within the ciliary compartment [3, 4]. Here, respiratory surface glycocalyx refers broadly to this glycan-rich interface, whereas glycocalyx layer refers more specifically to the membrane-proximal proteoglycan- and GAG-rich region. This terminology does not imply uniform bacterial entrapment or sequential movement through discrete layers.

The porcine respiratory surface contains functionally connected glycan-rich compartments. The mucus layer is enriched in gel-forming mucins such as MUC5AC and MUC5B, which contribute to mucociliary clearance. Recent biophysical analysis of porcine tracheal mucins indicates that these mucins form a relatively dense mesh network under airway-like conditions [25]. Beneath the mucus layer, the periciliary compartment contains tethered mucins, including MUC1, MUC4 and MUC16, together with keratan sulfate, contributing to the periciliary environment that supports ciliary function [26, 27]. Closer to the epithelial membrane, proteoglycans such as syndecans and glypicans carry GAG chains, including heparan sulfate (HS), chondroitin sulfate (CS) and dermatan sulfate (DS). Sulfation and carboxylation of these glycans contribute to a charged molecular environment capable of interacting with soluble mediators, extracellular matrix components and microbial ligands [8–12, 28].

This organization may influence Mhp attachment by regulating access to ciliary and membrane-proximal binding sites. Based on the ligand-binding properties of airway glycoconjugates, sulfated GAGs may provide potential sites for local bacterial enrichment, whereas mucins and the periciliary compartment may influence ligand accessibility. Because GAG-rich surfaces can influence ligand presentation and molecular accessibility [11, 29], these regions may provide a spatial context in which microbial adhesins and host-associated factors are brought into proximity. Importantly, the respiratory surface is not uniform but varies among airway regions and physiological conditions. Regional and physiological differences in mucin composition, distribution and glycosylation contribute to heterogeneity of the airway surface environment [8, 30, 31]. Porcine airway glycoprotein profiles also change during postnatal development, further illustrating that the respiratory glycan environment is dynamic [27]. In CFTR-deficient piglets, altered airway mucin sialylation is associated with impaired mucociliary clearance, demonstrating that changes in airway glycosylation can have functional consequences [32]. Although these variations have not been directly linked to Mhp colonization, they suggest that the glycan landscape encountered by Mhp may differ among hosts and airway microenvironments. Therefore, the glycocalyx-mediated framework proposed here should be interpreted as a conceptual model of the ciliated airway interface rather than a uniform representation of all respiratory.

Together, these components define a heterogeneous and interconnected respiratory interface in which Mhp encounters a glycan-rich environment rather than an exposed epithelial membrane. The conceptual organization of this interface and the potential regions involved in Mhp-host interactions are summarized in Figure 1.

**Figure 1:**
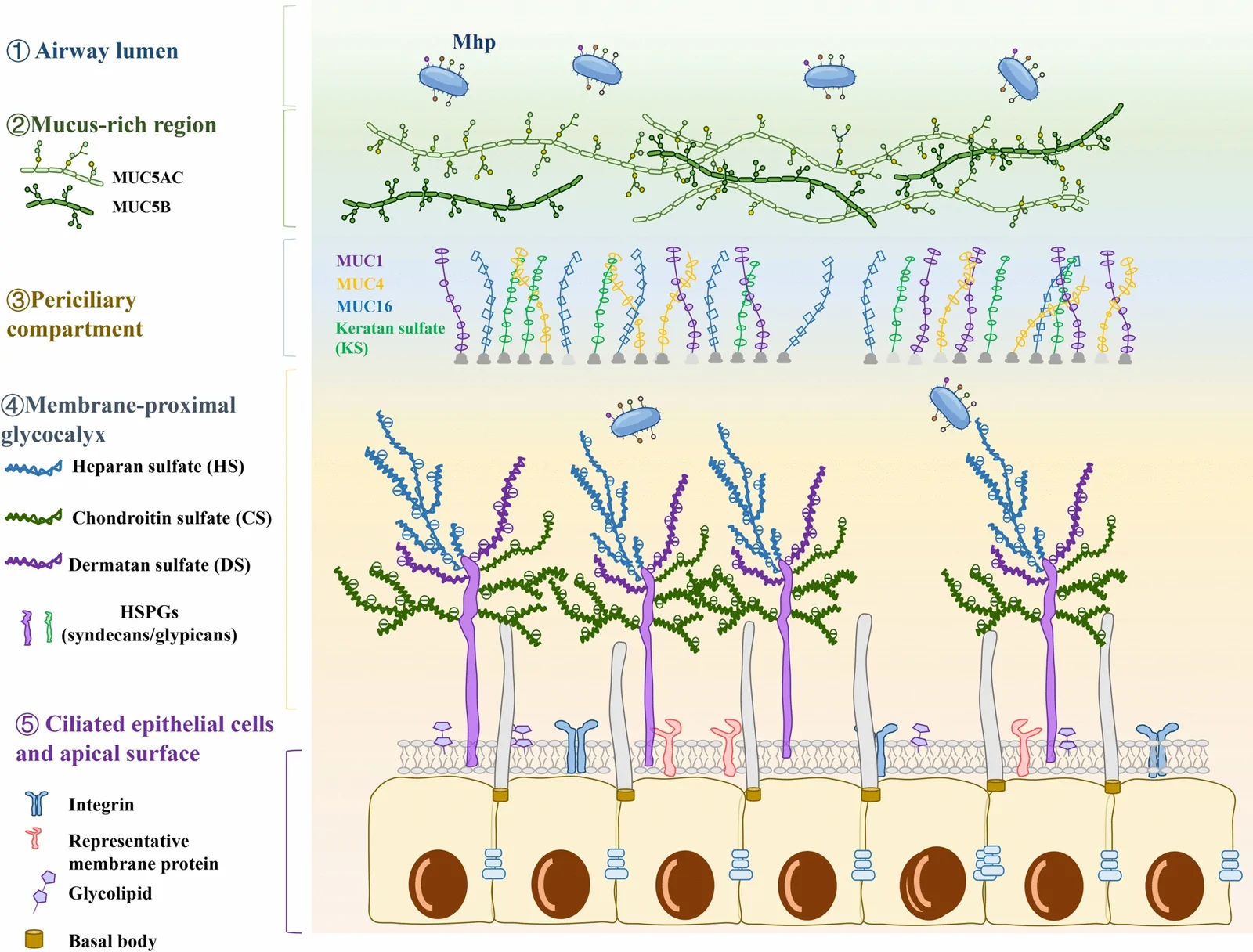
**Schematic organization of the heterogeneous porcine respiratory surface encountered by Mhp**. The figure illustrates major structural components of the ciliated airway interface, including a mucus-rich region containing the gel-forming mucins MUC5AC and MUC5B, a periciliary compartment containing membrane-tethered mucins and associated glycoconjugates, and a membrane-proximal glycocalyx containing proteoglycan-associated heparan sulfate, chondroitin sulfate and dermatan sulfate. These components are depicted as spatially interconnected and partially overlapping rather than as sharply separated anatomical layers. Mhp is shown at conceptual positions within the airway interface; in vivo ultrastructural studies have localized organisms at different positions within the ciliary compartment, but their distribution across the broader glycan-rich airway surface remains incompletely defined. The apical membrane and ciliary surfaces form a continuous cellular interface. Membrane-associated molecules are included as illustrative structural components and do not imply established receptor or signaling functions in Mhp infection. The figure is schematic and not drawn to scale.

## Mhp surface architecture and host-ligand binding: evidence for a multicomponent adhesion interface

The glycan-rich airway surface represents the host side of the Mhp adhesion interface, whereas the bacterial surface contains extracellular materials, fibrillar structures, adhesins and processed proteins that may collectively influence ligand presentation and host interaction.

### Capsule-like extracellular material and biofilm-associated organization

Early electron microscopy demonstrated that Mhp possesses extracellular structures beyond membrane-associated adhesins. Tajima et al. observed ruthenium-red-positive capsule-like material surrounding in vivo-passaged Mhp, together with fibrils connecting organisms and bridging the space between Mhp and host epithelial structures [3]. These structures were reduced after repeated in vitro passage, suggesting that extracellular organization may vary with physiological conditions. However, their molecular composition remains unresolved and they should not yet be considered a chemically defined conventional capsule. Consistent with this observation, comparative analyses have not identified a canonical polysaccharide synthase pathway in Mhp [33]. Mhp also forms biofilm-associated communities. Tassew et al. demonstrated strain-dependent biofilm formation on abiotic surfaces with extracellular matrix production and increased antimicrobial tolerance [34]. Raymond et al. further reported biofilm-like communities associated with porcine epithelial cells and experimentally infected pigs [35]. Biofilm-forming capacity also varies among strains and has been associated with virulence-related phenotypes, although this relationship does not establish causality [36]. Although the contribution of biofilms to early colonization remains unclear, capsule-like material, fibrillar structures and biofilm-associated matrices may influence bacterial aggregation, adhesin presentation and persistence at the respiratory interface.

### Multicomponent adhesin network and host-ligand binding

The P97/P102 paralogous protein family represents the best-characterized adhesion-associated system in Mhp. P97 contributes to ciliary attachment, and genetic disruption of P97 reduces cilia binding and epithelial-cell adherence [5, 6, 37]. Beyond ciliary interactions, P97/P102-family proteins can engage multiple host ligands. Two domains within P97 have been shown to bind heparin [14], whereas the R1 and R2 repeat regions of the P97 paralogue Mhp271 bind heparin, fibronectin and porcine cilia [15]. These findings support the ability of P97/P102-family proteins to interact with both ciliary structures and sulfated glycans. Current evidence supports GAG-binding and multiligand interactions, but does not yet establish structurally defined glycan-recognition mechanisms.

Proteolytic processing further expands Mhp surface functionality. The P159 precursor is cleaved into several surface-exposed fragments, including P27, P110 and P52. P159-derived regions have been shown to bind heparin and porcine cilia, indicating that proteolytic processing can generate additional host-interacting surface modules [18, 19, 38]. These processed products may increase the diversity and spatial distribution of ligand-binding activities on the Mhp surface, although their precise glycan specificity and contribution to native airway colonization remain unresolved.

Several additional proteins contribute to this multicomponent interface. Mhp384, Mhp385 and Mhp683 interact with GAGs and ciliary components [20, 21], whereas GAPDH and Mhp390 have been implicated in adhesion-associated Plg recruitment [24, 39]. Extracellular beta-actin has also been proposed as a supplemental host ligand based mainly on PK-15 cell models [23]. However, its contribution during early colonization of intact porcine respiratory epithelium remains unresolved.

Together, these findings support a multicomponent Mhp surface architecture in which adhesins, processed fragments and extracellular structures collectively influence interactions with cilia, glycans, extracellular matrix molecules and host factors. Direct evidence supports several individual ligand-binding activities, whereas the contribution of bacterial extracellular organization to native airway colonization remains incompletely defined.

## A glycocalyx-mediated infectious niche model for Mhp colonization

The evidence summarized above supports a model in which Mhp colonization involves coordinated interactions between bacterial surface molecules and the respiratory glycocalyx rather than a single adhesin-receptor event. In this framework, glycan-associated positioning, multivalent adhesin-ligand interactions and host-factor recruitment are considered interconnected components of the Mhp-airway interface. These interactions may overlap in space and time and should not be interpreted as a fixed sequential pathway. The glycocalyx is therefore considered not only a structural barrier but also a potential spatial platform that may influence ligand accessibility, bacterial positioning and local host–pathogen interactions.

### Glycan-associated enrichment and multivalent stabilization

Sulfated and carboxylated GAGs within the respiratory glycocalyx may contribute to Mhp association with the airway surface. Several Mhp adhesins and processed fragments have been shown to bind heparin or other defined GAGs [14, 15, 18–22]. More generally, GAG-protein interactions are influenced by structural features such as sulfation patterns, oligosaccharide composition and molecular conformation [29]. These observations support the possibility that sulfated glycans contribute to Mhp positioning at the airway interface, although the native GAG structures involved in porcine respiratory colonization remain incompletely characterized. Most direct evidence for Mhp-glycan interactions derives from heparin-binding assays or other defined GAG systems. The native respiratory environment is considerably more complex, with mucus movement, ciliary activity, spatially heterogeneous glycoconjugates and host-associated molecules potentially influencing bacterial attachment. Therefore, heparin binding should be interpreted as evidence of sulfated glycan-binding capacity rather than proof that identical interactions occur with the same specificity in vivo.

Within this interface, more stable attachment may arise from the combined contribution of multiple adhesin-ligand interactions. The P97/P102 family, P159-derived fragments, Mhp384, Mhp385, Mhp683 and other surface proteins provide overlapping ligand-binding activities that may support multivalent stabilization [14–22]. Although the physicochemical properties of charged glycoconjugates may influence molecular organization and ligand accessibility at the airway surface, specific adhesin-ligand interactions are currently better supported than a nonspecific charge-based mechanism. The contribution of Mhp surface physicochemical properties, including surface charge, remains poorly defined. Host and bacterial variation may further influence these interactions. Mucin composition and glycosylation patterns vary among respiratory regions and physiological or developmental conditions [27, 32, 40], potentially altering the host surface encountered by Mhp. Similarly, variation in adhesin sequences and repeat regions may contribute to strain-dependent differences in host interaction properties [7]. However, whether particular host glycan environments differentially influence attachment or colonization among Mhp strains remains unresolved.

An additional unresolved possibility is that transient interactions with GAG-rich regions may influence local bacterial retention or increase opportunities for subsequent engagement of other host-associated ligands. The presence of multiple GAG- or heparin-binding Mhp surface proteins provides a molecular basis for interactions with glycan-rich surfaces [14, 18–22], but does not demonstrate a dynamic retention mechanism. Direct evidence showing GAG-dependent changes in Mhp residence time, repeated binding behavior or subsequent ligand engagement on native porcine airway surfaces is currently lacking. Therefore, any contribution of transient GAG-associated interactions to local retention should be regarded as a hypothesis requiring direct experimental validation rather than an established step in early colonization.

### Plasminogen recruitment and potential local interface modification

At the Mhp-airway interface, recruitment of host factors may contribute to functional modification of the local adhesion environment. Among these host factors, Plg recruitment represents one of the better-supported molecular connections between Mhp surface interactions and potential extracellular matrix-associated effects. Multiple Mhp surface proteins, including P102-related proteins, processed adhesins, Mhp107, P146, GAPDH and Mhp390, have been associated with Plg binding or Plg-dependent functions [13, 16, 17, 22, 24, 39]. Plg binding is only the first step in this proposed functional link; downstream effects would require conversion of surface-associated Plg to active plasmin. Whether such activation occurs at Mhp adhesion sites under physiological conditions remains unresolved. If local plasmin generation occurs, it may promote degradation of extracellular matrix components, including fibronectin and laminin, and contribute to epithelial barrier disruption. In the context of the glycocalyx-mediated model, GAG-rich regions may provide a spatial platform where Mhp adhesins, host glycans, extracellular matrix molecules and Plg become locally concentrated, thereby linking surface attachment with localized proteolytic activity.

Plasmin may also influence complement activity [41, 42], although this has not been demonstrated at Mhp adhesion sites in vivo. Mhp additionally uses Plg-independent complement evasion mechanisms, including EF-Tu-mediated factor H binding [43]. Therefore, Plg recruitment should be considered one component of a broader host-interaction strategy rather than the sole mechanism of immune modulation. Importantly, direct evidence demonstrating plasmin-mediated extracellular matrix remodeling or complement modulation at Mhp adhesion sites in infected porcine airway tissues remains unavailable.

Overall, the glycocalyx-mediated infectious niche model integrates glycan-associated positioning and possible local retention, multivalent adhesin-ligand interactions and host-factor recruitment as interconnected, potentially overlapping components of the early Mhp-airway interface. Their spatial and temporal relationships remain to be established experimentally and are not intended to represent a fixed sequence of colonization events. This integrated working model is summarized in Figure 2. The evidence status, unresolved questions and priority validation approaches for major components of this model are summarized in Table 1.

**Figure 2:**
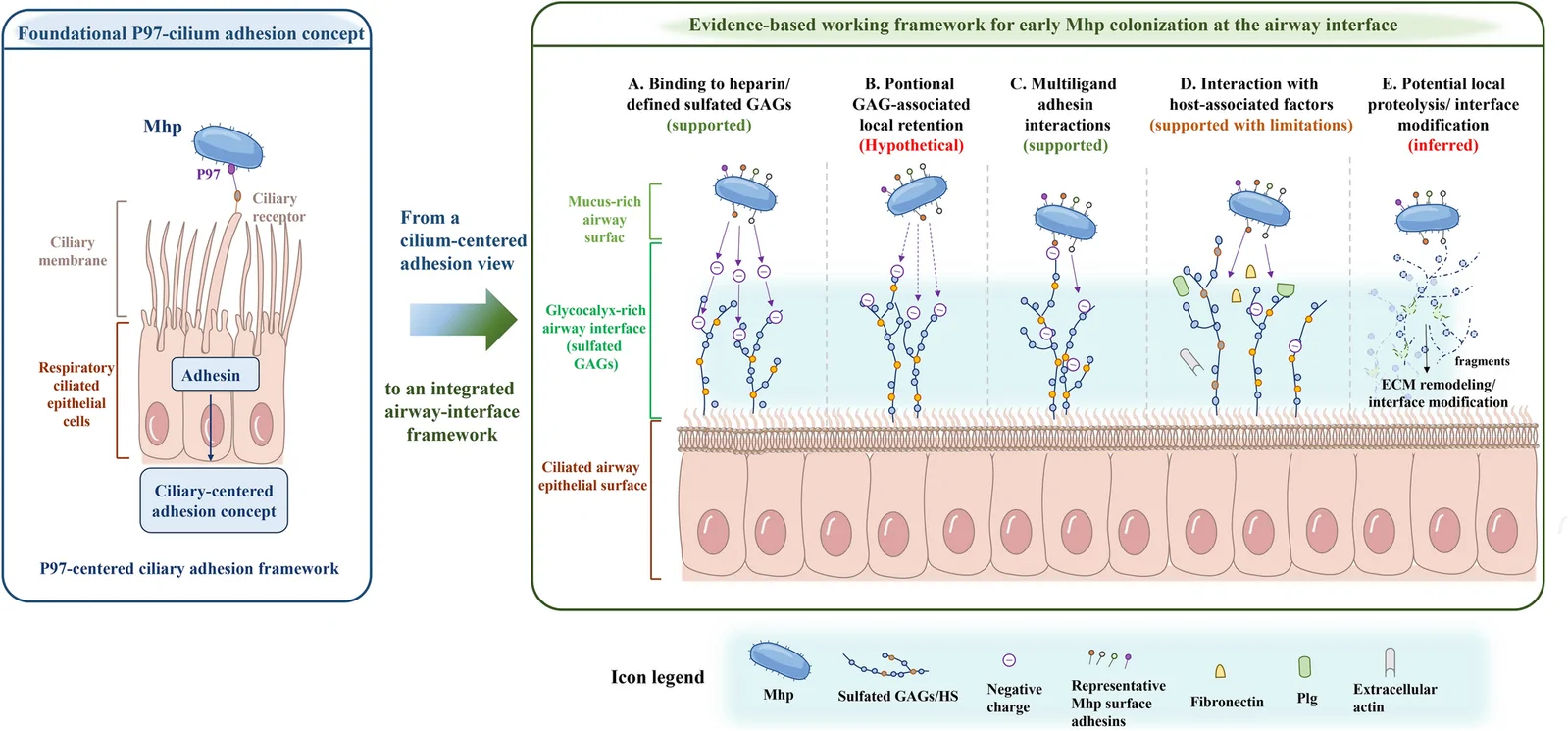
**Evidence-based working framework for early Mhp colonization at the porcine airway interface**. The left panel summarizes the foundational P97-cilium adhesion concept, whereas the right panel integrates current evidence and unresolved questions concerning Mhp interactions with the glycan-rich ciliated airway surface. The framework includes binding to heparin or defined sulfated GAGs, potential GAG-associated local retention, multiligand adhesin interactions, interactions with host-associated factors such as fibronectin and Plg, and potential local proteolysis or interface modification. Direct molecular evidence supports Mhp binding to heparin or defined sulfated GAGs and the ability of multiple surface proteins to interact with host ligands, whereas GAG-associated local retention remains hypothetical. Interactions with host-associated factors are supported but remain incompletely validated in native airway tissues, and downstream proteolytic or interface-modifying effects are inferred and require further confirmation. The illustrated components may overlap spatially and temporally and are not intended to represent a fixed or obligatory sequence of colonization events. Solid elements indicate interactions supported by current evidence, whereas dashed elements indicate hypothetical or inferred processes requiring further validation.

**Table 1:** **Evidence status and validation priorities for the glycocalyx-mediated Mhp infectious niche model**

Model component	Evidence status	Key unresolved issue	Priority validation
Binding to heparin/defined sulfated GAGs	Supported	Native porcine airway GAG ligands and binding specificity remain unclear	Defined GAG assays; glycan arrays; GAG perturbation
Multiligand adhesin interactions	Supported	Contribution of individual adhesins to native airway colonization remains unclear	Adhesin-deficient mutants; porcine air–liquid interface cultures; ex vivo explants
Bacterial extracellular organization	Supported with limitations	Roles of capsule-like material and biofilm-associated matrices in early airway attachment remain unclear	In vivo imaging; structural characterization
Potential GAG-associated local retention	Hypothesis	Whether GAG interactions alter Mhp retention or subsequent ligand engagement is unknown	Single-bacterium tracking; shear-flow assays; GAG perturbation
Host glycan-dependent variation in attachment	Hypothesis	Links among airway glycan variation, strain-specific binding and colonization remain untested	Airway glycomics; strain-specific binding; ex vivo/in vivo correlation
Plg recruitment and potential local proteolysis	Supported with limitations	Local Plg activation and causal effects at airway adhesion sites remain unresolved	Plg blockade; plasmin probes; ECM cleavage assays
Potential glycocalyx remodeling	Exploratory hypothesis	Whether Mhp alters or exploits infection-associated glycan changes is unknown	Glycomics; transcriptomics; GAG profiling; spatial analysis

Supported indicates direct molecular or functional evidence. Supported with limitations indicates direct evidence with limited spatial or causal validation in native airway tissues. Hypothesis indicates a testable mechanism inferred from available evidence. Exploratory hypothesis indicates a biologically plausible extension with limited direct support.

## Knowledge gaps and future experimental priorities

The glycocalyx-mediated infectious niche model integrates direct molecular evidence, mechanistic inference and unresolved questions. Current evidence supports interactions between Mhp surface proteins and sulfated GAGs, cilia, fibronectin and Plg, whereas several mechanistic connections involved in early colonization remain incompletely understood. This section therefore summarizes key knowledge gaps and experimental priorities required to validate or refine the proposed framework.

### Unresolved questions regarding glycocalyx involvement during early Mhp colonization

Whether Mhp modifies the respiratory glycocalyx during infection remains unknown, as direct evidence for changes in airway glycan composition, GAG sulfation, mucin glycosylation or related host pathways is currently lacking. Nevertheless, this question is relevant because the respiratory glycocalyx is a dynamic structure influenced by inflammation, epithelial stress and tissue injury [28, 30]. Changes in mucin organization, GAG abundance or glycan accessibility could potentially modify the host binding environment encountered by Mhp.

If glycocalyx alterations occur, whether they arise from host responses or bacterial activities remains unresolved. Future studies using porcine airway air-liquid interface cultures, ex vivo respiratory tissues and infected animals should integrate transcriptomic analysis, glycomics, GAG profiling and spatial approaches to determine whether Mhp exploits a pre-existing glycan landscape or influences host surface organization during infection.

### Anti-adhesion strategies as future directions

Viewing Mhp colonization as a glycocalyx-associated adhesion process suggests that anti-adhesion approaches may provide complementary strategies to existing control measures. If sulfated glycans and multivalent adhesin interactions contribute to early Mhp attachment, interfering with these interactions could potentially reduce bacterial association or attachment without directly targeting bacterial viability.

One possible approach is the development of defined glycan-based competitors or engineered molecules that interfere with Mhp-glycan interactions. However, because host GAGs participate in normal ligand presentation and epithelial homeostasis [11, 29], selective targeting of Mhp-associated interactions will be required. A second approach is to target conserved adhesin regions or processed surface fragments involved in multivalent attachment. Such strategies will need to consider adhesin paralogy, processing patterns and strain variation. In addition, Plg-associated interactions represent a potential target area, although their precise contribution to the earliest stages of colonization requires further clarification.

Overall, anti-adhesion strategies should be evaluated only after the key components of the glycocalyx-mediated model have been validated in physiologically relevant systems, including porcine airway models, ex vivo tissues and in vivo colonization studies.

## Conclusions and perspectives

Current evidence supports a broader view of Mhp colonization as a multicomponent interaction between bacterial surface molecules and the glycan-rich respiratory interface. The P97-centered adhesion framework remains foundational for understanding ciliary attachment, but subsequent studies have identified multiple Mhp surface proteins and host ligands, including sulfated GAGs, fibronectin, Plg and extracellular actin. Together, these findings indicate that early colonization is likely to involve coordinated interactions within the ciliated airway environment rather than a single adhesin-receptor event.

The glycocalyx-mediated infectious niche model proposed here integrates glycan-associated positioning, multivalent adhesin-ligand interactions and host-factor recruitment as interconnected components of the Mhp-airway interface. However, their spatial and temporal relationships remain incompletely resolved, and the model should not be interpreted as a fixed sequence of colonization events. In particular, the contribution of transient GAG-associated interactions to local retention, the influence of host glycan variation on strain-dependent attachment, and possible glycocalyx remodeling remain unresolved. Plg recruitment is comparatively well supported at the molecular level, but whether it leads to localized plasmin activity and interface modification in infected porcine airway tissues requires direct validation.

Future studies using physiologically relevant porcine airway models, ex vivo tissues and in vivo approaches will be essential for defining which glycocalyx-associated interactions are functionally important during early colonization. Such validation will determine whether specific glycan-adhesin or host-factor interactions can be exploited as complementary anti-adhesion targets for the control of porcine enzootic pneumonia.

## Data Availability

No datasets were generated or analysed during the current study.

## Abbreviations

Mhp: *Mycoplasma hyopneumoniae*
GAGs: glycosaminoglycans
Plg: plasminogen
MUC5AC: mucin 5AC
MUC5B: mucin 5B
MUC1: mucin 1
MUC4: mucin 4
MUC16: mucin 16
HS: heparan sulfate
CS: chondroitin sulfate
DS: dermatan sulfate
KS: keratan sulfate
HSPGs: heparan sulfate proteoglycans
ECM: extracellular matrix
